# Association between ambient air pollution exposure and insomnia among adults in Taipei City

**DOI:** 10.1038/s41598-022-21964-0

**Published:** 2022-11-09

**Authors:** Liang-Ju Tsai, Tzu-Hsuen Yuan, Ruei-Hao Shie, Ching-Han Chiang, Chang-Chuan Chan

**Affiliations:** 1 Institute of Environmental and Occupational Health Sciences, College of Public Health, National Taiwan University, No.17, Xu-Zhou Rd., Taipei, 10055 Taiwan; 2 Department of Family Medicine, Taipei City Hospital, Renai Branch, Taipei, Taiwan; 3 Department of Health and Welfare, College of City Management, University of Taipei, Taipei, Taiwan; 4 Green Energy and Environment Research Laboratories, Industrial Technology Research Institute, Hsinchu, Taiwan

**Keywords:** Environmental impact, Public health

## Abstract

Ambient air pollution was known to cause central nervous system diseases and depressive symptoms. In this study, we examined the associations between air pollution exposure and the prevalence of insomnia in Taipei City of Taiwan. We applied the health information system of electrical medical records of Taipei City Hospital to collect a total of 5108 study subjects (insomniacs N = 912 and non-insomniacs N = 4196) over 18 years old from the family medicine and internal medicine outpatients of six branches of Taipei City Hospital. These patients were grouped into insomniacs and non-insomniacs following the primary insomnia diagnosis (ICD9:780.52, 780.54, 307.41, 307.42, ICD10: G47.00, G47.01, G47.09, F51.01, F51.09) and the prescription times of anxiolytics and hypnotics. We estimated one-year average concentrations of PM_2.5_, ozone, and NO_x_ before the first date of insomnia diagnosis and the last date of outpatient visit for insomniacs and non-insomniacs, respectively, by using the data of nearest air quality monitoring stations relative to study subjects’ residential addresses. Logistic regression analysis was employed to examine the independent effects of air pollution concentrations on the risk of insomnia. One-year average PM_2.5_, ozone, and NO_x_ levels for insomniacs was significantly higher than those of non-insomniacs. After adjusting for confounding factors, increase each 1(μg/m^3^) in one-year average PM_2.5_ showed a statistically significant association with insomnia (the odds ratio 1.610, 95% CI [1.562,1.660]). As to multi pollutants, one-year average PM_2.5_ (1.624, [1.570, 1.681] and ozone (1.198, [1.094, 1.311]) exposure showed a significant association with insomnia. Subgroup analysis revealed that the influence of PM_2.5_ and ozone on insomnia have significant risks in people with major chronic disease. This study demonstrated a positive association between PM_2.5_ and ozone exposure and the prevalence of hypnotic-treated insomnia. Especially, the people with major chronic diseases were with obvious effect of PM_2.5_ and ozone on risk of insomnia.

## Introduction

### Air pollution’s health effect

Common air pollution includes particulate matter (often referred to as particle pollution), ozone, oxocarbon, sulfur oxides, nitrogen oxides, and these pollutants were reported to be the leading contributors to many diseases and death^1^. Ambient air pollution was found to be associated with ischemic heart diseases, cerebrovascular diseases, chronic obstructive pulmonary disease, lower respiratory tract infections, lung cancer deaths and chronic kidney disease^2,3^. Regarded the effect of air pollution on the central nervous system, the risk of Alzheimer’s disease, depressive symptoms in the elderly increased with exposure to PM_2.5_ and NO_2_^4,5^. Currently, the air pollution due to PM_2.5_ was considered as a critical problem threatening human health because it can easily penetrate into the deeper lung regions^6^. Exposure to PM_2.5_ could endanger multiple organs in the body, and even led to systemic adverse effects^7^. For epidemiological studies, Ambient PM_2.5_ was the fifth-ranking mortality risk factor in 2015^2^. Ambient air pollution, mostly by PM_2.5_ was estimated to cause 3.3 million premature deaths per year worldwide, predominantly in Asia^8^. Since past evidence indicated that PM_2.5_ was related to health risks, it become of increasing public concern in the world.

### Insomnia

Insomnia was an important public health issue with a significant negative impact on individuals' physical and social performance, ability to work and quality of life, as well as on society as a whole^9^. Common causes include stress, an irregular sleep schedule, poor sleeping habits, mental health disorders like anxiety and depression, physical illnesses and pain, medications, neurological problems, and specific sleep disorders. There were many literatures reporting the relationship between CKD and neurological/mental diseases. The diseases may affect sleep quality bidirectionally^10,11^ (Stephanie C Maung et al., 2016) (Kirstie N Anderson et al., 2013). -Insomnia is usually described as a self-reported symptom with sleep difficulty. The prevalence of sleep disturbance varied from 5 to 40% based on the different definitions in the literature^12–14^. A previous study showed that 11.14% of elderly outpatients were diagnosed with insomnia in 2001 in Taiwan^15^. Insomnia status predicted poor psychological well-being even after controlling for sociodemographic factors and health status^16^. Among individuals with chronic insomnia, 34.7% had attempted to seek professional help and 23.5% had used hypnotics to address symptoms^17,18^. Use of these drugs may cause problems of addiction, psychomotor retardation, memory impairment, paradoxical disinhibition, depression, and emotional blunting^19^. Therefore, it is an important issue to find out the key risk factors for insomnia.

### Air pollution and sleep

Sleep is a complex process modulated by neurotransmitters in the central nervous system, so we were curious about whether sleep is affected by air pollutants, similar to Alzheimer’s disease and emotional expression. PM_2.5_ can reach the lungs, and be transported into the blood and the brain^20^. PM_2.5_ transported to the brain may thus lead to neural inflammation^21^, which may also contribute to the disturbance of the sleep–wake cycle^22,23^.

One multi-center study in the United States found that limited increases in apnea and hypopnea and decreases in sleep efficiency are associated with increases in short-term variations in PM_10_^24^. A previous study found that exposure to heavy metals in metal fume PM_2.5_ may disturb sleep among welding workers due to chronic hypoxia from decreased pulmonary function^25^. However, only metal fume PM_2.5_ was investigated in this study. Whether the effect of general ambient PM_2.5_ exposures on sleep is consistent with the effect of metal fume exposure remains unknown. Another recent study with children used the Sleep Disturbance Scale for Children as a tool to evaluated sleep found 4-year exposure to PM_1_ and PM_2.5_ air pollutants increases the odds of sleep disorder in children^26^. Short-term variations in ozone concentration had to do with SDB (sleep disordered breathing)^27^. A past study said that long-term NO_2_ exposure was related to poor sleep quality^28^. Previous studies have focused primarily on the effect of air pollution on sleep-related respiratory symptoms, only a few studies have examined the relationship between air pollution and insomnia.

### Urban

Many studies showed that living at a fast and busy pace in the city can cause insomnia. Children who lived in urban appeared to have sleeping problems more than children who lived in a rural setting^29^. Urbanization was statistically significantly associated with insomnia^30^. Non-urban residents slept better than those in the Tokyo metropolitan area^31^. The problems of PM_2.5_ usually were serious in urban and it induced much more mortality than those in rural areas^32^. Air pollution exposure and insomnia are serious issues in urbanization. Therefore, our study aimed to explore the relationship between insomnia and ambient air pollution in urban.

### Objectives

According to the above studies and the results we have observed, this study aimed to examine the associations between one-year average PM_2.5_, ozone, and NO_x_ exposure and medical doctor-diagnosed, hypnotic-prescribed insomnia in an adult population. We also compared the effect of these air pollutants on insomnia in subjects with or without major chronic diseases in Taipei City.

## Methods

### Study subjects

We conducted an observational study from January 1, 2014 to December 31, 2016 using a health information system (HIS) of electrical medical records from Taipei City Hospital. The first outpatient visit was restricted to the one during the observation period (January 1, 2014 to December 31, 2016). This study involved 55,481 outpatients of the internal medicine department of Songde Branch, Zhongxiao Branch, Yangming Branch, Heping Fuyou Branch, Renai Branch, and Zhongxing Branch of Taipei City Hospital between January 1, 2014 and June 30, 2014. After excluding patients under 18 years old, a total of 55,183 patients remained.

**Figure 1:**
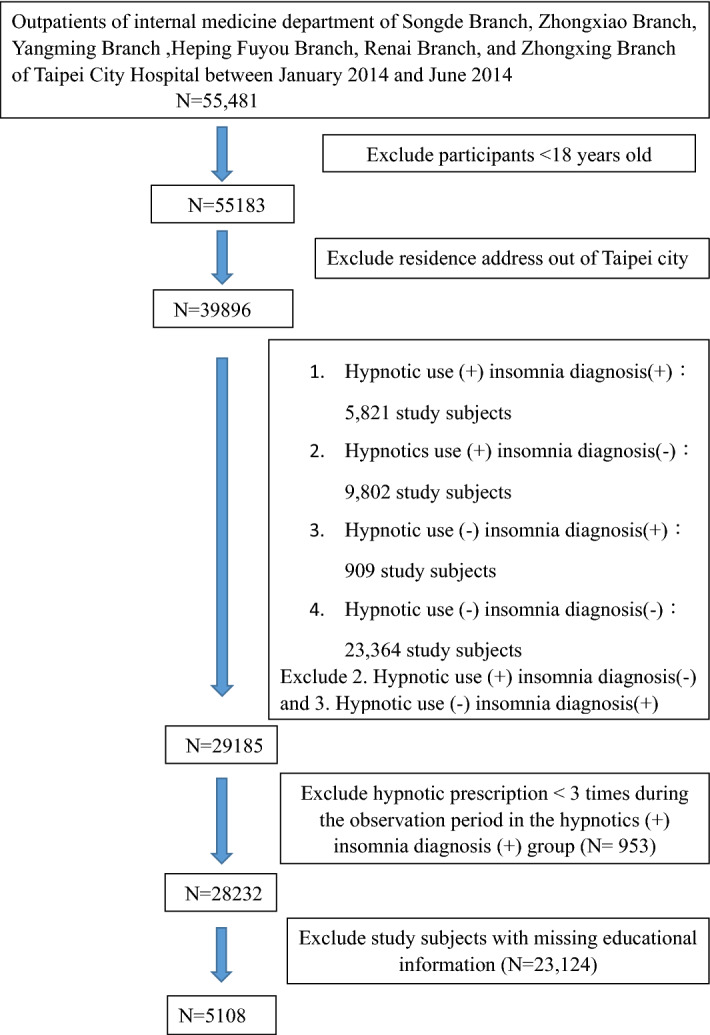
Flow chart of enrollment.

Patients whose residential address was beyond 12 districts (Zhongzheng district, Datong district, Zhongshan district, Songshan district, Daan district, Wanhua district, Xinyi district, Shilin district, Beitou district, Neihu district, Nangang district, and Wenshan district) of Taipei City were further excluded, and 39,896 patients were left. These 39,896 participants were further grouped into one of four categories based on the prescriptions and International Statistical Classification of Diseases and Related Health Problems (ICD) diagnosis record from their first outpatient clinic visit to December 31, 2016 as follows: (1) prescription of hypnotics at least once and with at least one record in the ICD codes of primary insomnia; (2) prescription of hypnotics at least once in the absence of insomnia diagnosis based on ICD codes; (3) no prescription of any hypnotic prescription but with at least one insomnia diagnosis based on ICD codes; and (4) no prescription of any hypnotic prescription and also no insomnia diagnosis based on ICD codes. We excluded those who were prescribed with hypnotics in the absence of insomnia diagnosis and those who never used hypnotics but were diagnosed with insomnia based on ICD codes. A total of 29,185 participants remained. We then excluded those who were prescribed with hypnotics less than three times, and 28,232 participants were left. Participants with incomplete educational information were excluded, and the remaining 5108 study subjects were enrolled into further analysis (Fig. 1). Major chronic diseases are risk factors of insomnia. People with major chronic diseases are more likely to have insomnia, so we selected some diseases whose common symptoms may affect sleep directly or indirectly, including mental diseases that were highly related to insomnia symptoms. (see supplemental material, ICD-9 and ICD-10 codes of major chronic diseases). These 5108 study subjects were considered as having any one of the above major chronic diseases or having none of the above-mentioned major chronic diseases. For those without any of the above diseases, the main disease of these participants is shown in the Supplemental material, Diseases of the study subjects without major chronic diseases (N = 1069).

### Definition of insomnia

Patients were dichotomized to the insomniac and non-insomniac groups according to the ICD codes of primary insomnia diagnosis and the prescription of anxiolytics and hypnotics during the observation period. The ICD codes were used to define the insomnia diagnosis. Patients given any of the following ICD codes (ICD9:780.52, 780.54, 307.41, 307.42, ICD10: G47.00, G47.01, G47.09, F51.01, F51.09) with anxiolytics and hypnotics being prescribed for at least three times during any time from the first outpatient visit to December 31, 2016 were recognized as insomniacs. Patients with neither hypnotic prescription nor insomnia diagnosis based on ICD codes during the study period were defined as non-insomniacs. Study anxiolytics and hypnotic drugs included benzodiazepines and Z drugs (See supplementary material, Study anxiolytics and hypnotic drugs and defined daily dose (DDD)). Use was quantified from the entry point to December 31, 2016 as the defined daily dose (DDD). The DDD is assumed as the average maintenance dose per day for a drug used for its main indications in adults (considered to be someone of 70 kg in body weight)^33^. We ascertained the diagnosis code and receipt of hypnotic and anxiolytic drugs from HIS electronic medical records of Taipei City Hospital.

### Air pollution exposure

We estimated the individual concentrations of average air pollutants PM_2.5_, ozone, and NO_x_ exposure by using the nearest air quality monitoring station database established by the Taiwan Environmental Protection Administration. We used a geographic information system (GIS; arc GIS version 10.1) to locate the 5108 participants’ residential addresses retrieved from HIS medical records and the 10 air quality monitoring sites around Taipei City and New Taipei City on separate maps first. We then used the “near” analytic function in ArcGIS to obtain the nearest monitoring station and the distance between the station and every residential address.

**Figure 2:**
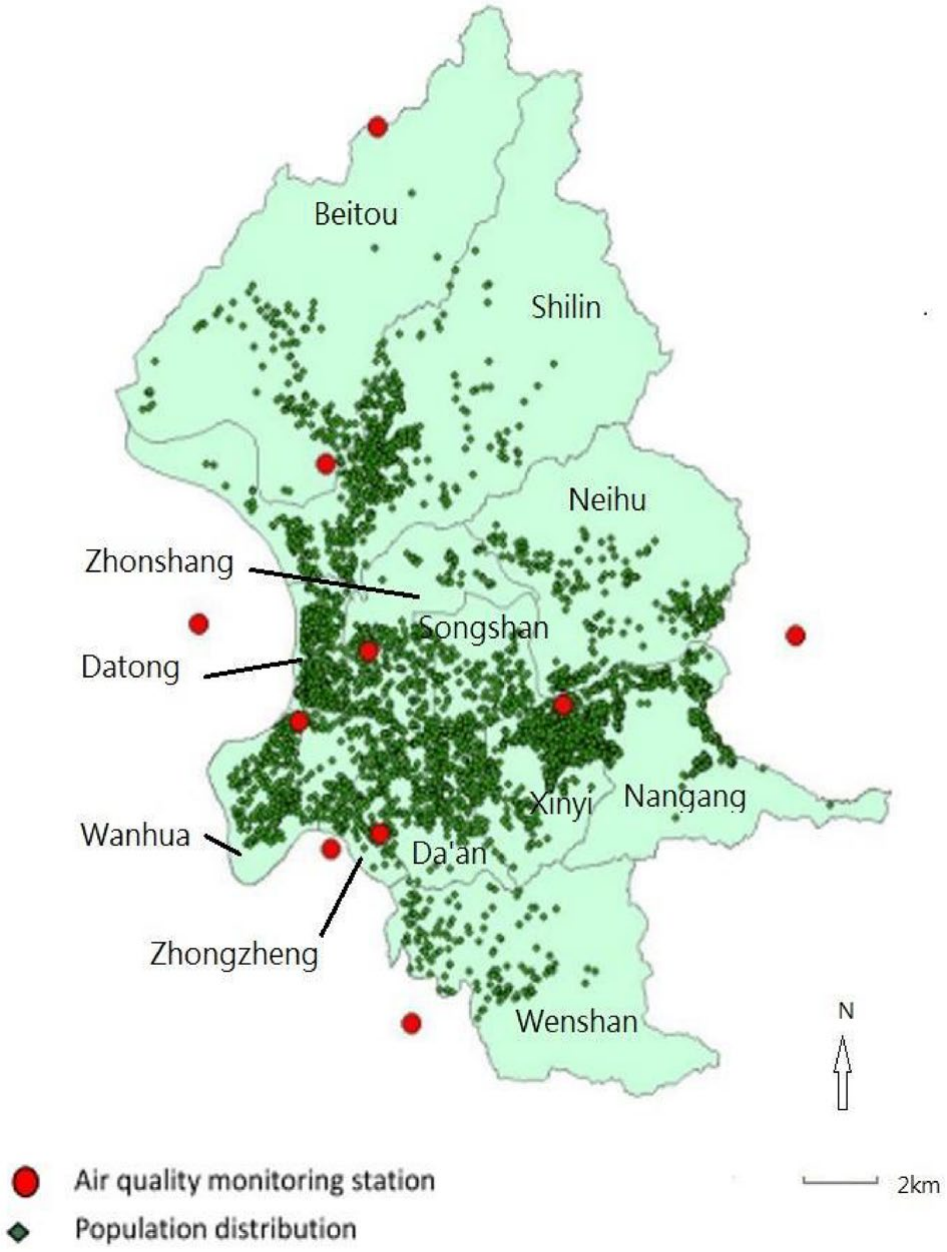
Residential addresses of the 5108 study subjects. The figure was generated by ArcGIS version 10.5 (URL link: https://www.esri.com/en-us/arcgis/about-arcgis/overview).

The distribution of the 5108 residential addresses and their relative positions with the 10 air quality monitoring sites are illustrated in Fig. 2. The median distance between air quality monitoring site and subjects’ residential address was 1537.1 m (first–third quartiles, 1011.8–2267.9 m).

Exposure for insomniacs dated back to 1 year before the first date of insomnia diagnosis. Exposure for non-insomniacs dated back to 1 year before the last date of the outpatient visit. For each patient, we used hourly recorded PM_2.5_, ozone, and NO_x_ data from the nearest air quality monitoring station relative to their living residence at the time of recruitment to calculate the average ambient air pollution level of a certain time period before insomnia diagnosis. For example, if a subject’s diagnosis day in the electrical medical record was June 1, 2015, the one-year exposure was the mean of the hourly air pollution concentrations from June 1, 2014 (12:00) to June 1, 2015 (12:00). Air pollution exposure is a continuous variable in this study.

### Measurement of potential confounding factors

Age, gender, educational level (below college [non-college graduates] or above college educational level [college graduates]), noise-related variables, major chronic diseases (as described in Section *Study subjects*) were adjusted for insomnia. Residence floor information retrieved from HIS medical records and roadway proximity were considered noise indicators.

We categorized participants’ residence floor into one and two, three and four, or above five floors. Road area (m^2^) and major road area (m^2^) within 50 and 100 m of circular buffer radii within each residential address and elevated road length (m) within 100 m of circular buffer radii around each residential address were calculated with arc GIS version 10.1. Major roads in Taipei City were wider than 20 m and contained more than dual carriageways.

Continuous variables included age and noise-related variables. Categorized variables included gender, educational level, and major chronic diseases. Information of age, gender, and educational level was retrieved from HIS medical records.

### Statistical analysis

Study subjects were dichotomized as insomniacs and non-insomniacs. Chi-square test and t-test were used to compare the basic characteristics and environmental factors between insomniacs and non-insomniacs. We used multiple logistic regression analyses to examine the odds ratio and 95% confidence interval (CI) between air pollution concentrations and insomnia incidence. Insomnia incidence is a binominal outcome. The confounders included in multiple regression analysis were based on the review of insomnia risk factors and the common air pollutants that may confound the effect of PM_2.5_ on insomnia (described in “Measurement of potential confounding factors” section). We used two adjusted (multiple analysis) models (single-pollutant and multiple-pollutants models) to estimate the risk of air pollutants on insomnia. Single-pollutant model adjusted for significant risk factors for insomnia, whereas multiple-pollutants model adjusted for ambient air pollution NO_x_ and O_3_ besides other risk factors. Stratified analysis was conducted to analyze the effects of PM_2.5_, ozone, and NO_x_ exposure in subgroups with major chronic diseases and subgroups without major chronic diseases, respectively.

All of the analyses were performed with SAS software (Version 9.4).

### Ethical approval

This study was approved by the Institutional Review Board of the Taipei City Hospital [TCHIRB-10603103-E]. Since this is a retrospective study, the informed consents were waived by the Institutional Review Board of the Taipei City Hospital. In addition, this study was performed in accordance with the Declaration of Helsinki and approved by an appropriate ethics committee.

## Results

The comparison of age, gender, educational information, and residence floor information between the insomniacs and non-insomniacs with complete educational information (28,232 subjects) is shown in Supplemental material, Comparison of age, gender, and education of the insomniacs and non-insomniacs of the outpatients aged above 18 years old and living in Taipei City (N = 28,232). A significant difference was observed in terms of age, gender, and educational level between the two groups. The comparison of age, gender, and insomnia status between 5108 cases (complete educational information) and the excluded 23,124 cases (missing educational information) from 28,232 cases is shown in Supplemental material, Comparison of age, gender, and insomnia status between 5108 cases with complete educational information and excluded 23,124 cases with missing educational information.

Age of the cases with complete educational information was statistically younger than that of the cases with missing educational information (61.33 years old vs. 63.08 years old). Regarding gender distribution, cases with complete educational information were statistically less female than those with missing educational information. The ratio of insomniacs and non-insomniacs was not significantly different between the cases with and without educational information.

Table 1 shows the comparison of age, gender, major chronic diseases, educational level, residence floor level, road area, major road area, elevated road length with radii of 50 and 100 m around the residential address between non-insomniacs and insomniacs of the final 5108 outpatients in Taipei City Hospital. The study population consisted of 82.15% of non-insomniacs and 17.85% of insomniacs. Our study subjects consisted of older residents compared with the general population. Insomniacs were composed of older individuals and more female cases than non-insomniacs. The mean age was 60.4 years for the non-insomniacs and 65.64 years for the insomniacs. Approximately 53.35% of the insomniacs were female, whereas 45.42% of the non-insomniacs were female. The number of insomniacs with chronic diseases was significantly higher than that of non-insomniacs (86.95% vs. 59.63%). There were significantly more non-insomniacs with educational level higher than college compared with non-insomniacs (28.68% vs. 18.53%).

**Table 1:** Comparison of age, gender, major chronic diseases, educational level, residence floor, road area, major road, elevated road and distribution of exposure concentrations of PM_2.5_ (unit: µg/m^3^), ozone (unit: ppb), and NO_x_ (unit: ppb) at 1 year before the date of insomnia diagnosis for insomniacs or last date of outpatient visit for non-insomniacs based on monitoring stations between non-insomniacs and insomniacs of 5108 outpatients in Taipei City Hospital.

	Non-insomniacs	Insomniacs	Total	*P* value
Number of patients (%)	4196 (82.15)	912 (17.85)	5108	
**Age, year**				
Mean (SD)	60.40 (15.32)	65.64 (13.58)	61.33 (15.16)	< 0.0001
Gender				< 0.0001
Female, n (%)	1906 (45)	487 (53)	2393 (47)	
Male, n (%)	2290 (55)	425 (47)	2715 (53)	
Major chronic disease*				< 0.0001
No, n (%)	1026 (96)	3170 (78)	4196 (82)	
Yes, n (%)	43 (4)	869 (22)	912 (18)	
Education level, n (%)				< 0.0001
Non-college graduates	2993(71)	743 (81)	3736 (73)	
College graduates	1203 (29)	169 (19)	1372 (27)	
Residence floor, n (%)				0.7256
1 and 2	1120 (32)	227 (30)	1347 (31)	
3 and 4	1338 (38)	283 (38)	1621 (38)	
≥ 5	1096 (31)	241 (32)	1337 (31)	
**Road area, m**^**2**^				
Buffer 50 m, mean (SD)	1755 (1,151.4)	1852.6 (1,061.4)	1772.4 (1136.4)	0.0192
Buffer 100 m, mean (SD)	8672.7 (2,757.3)	8724.6 (2,775.6)	8681.9 (2760.3)	0.6082
**Major road, m**^**2**^				
Buffer 50 m, mean (SD)	162.1 (570.6)	143.2 (512.2)	158.8 (560.6)	0.3571
Buffer 100 m, mean (SD)	849.7 (1,962.5)	883.9 (1,992.1)	855.9 (1,967.7)	0.6352
**Elevated road, m**				
Buffer 100 m, mean (SD)	1727.6 (6,553.5)	1993.4 (6,839.5)	1757.1 (6,604.9)	0.4937
1 year PM_2.5_ Mean ± SD (min–max)	18.7 ± 3.4 (10.6–32.2)	25.5 ± 3.4 (12.8–32.2)		< 0.0001
1 year O_3_ Mean ± SD (min–max)	25.3 ± 2.8 (20.8–42.5)	26.0 ± 3.6 (20.8–42.2)		< 0.0001
1 year NO_x_ Mean ± SD (min–max)	27.7 ± 5.8 (3.9–39.5)	28.6 ± 6.3 (4.3–39.5)		0.009

*Chronic diseases include psychiatric disease, coronary heart disease, congestive heart failure, cerebrovascular disease, dementia, pulmonary disease, peptic ulcer disease and reflux esophagitis, chronic hepatic disease, diabetes, chronic kidney disease, and malignancy.

Table 1 also shows the distribution of particulate matter (2.5 μm or less in aerodynamic diameter [PM_2.5_]), ozone, and NO_x_ exposure concentrations of 1 year before the date of insomnia diagnosis for insomniacs and last date of outpatient visit for non-insomniacs based on monitoring stations. The average PM_2.5_ levels of 1 year for insomniacs were 25.5 µg/m^3^. The average PM_2.5_ levels of 1 year for non-insomniacs were 18.7 µg/m^3^. Both of the values exceeded the National Ambient Air Quality Standards of Taiwan for PM_2.5_ (15 µg/m^3^). The average ozone levels of 1 year for insomniacs were 26.0 ppb. The average ozone levels of 1 year for non-insomniacs were 25.3 ppb. The average NO_x_ levels of 1 year for insomniacs were 28.6 ppb. The average NO_x_ levels of 1 year for non-insomniacs were 27.7 ppb.

Table 2 shows the associations of single pollutant PM_2.5_ and multi-pollutants with insomnia after adjusting for age, gender, educational level, major chronic diseases, radius of 50 m of road area, and residence floor. Every 1 year increase in age demonstrated a significantly positive association with insomnia (odds ratio 1.020, 95% CI [1.012–1.028]). Patients with chronic major diseases had significantly higher risk on insomnia than patients without chronic major diseases (odds ratio 31.31, 95% CI [19.83–49.42]). Regarding educational level, non-college graduates had higher risk on insomnia than college graduates (odds ratio 1.608, 95% CI [1.201–2.154]).Every increment of 1(μg/m^3^) in one-year mean PM_2.5_ exposure demonstrated a significantly positive association with insomnia(odds ratio 1.610, 95% CI [1.562–1.660]). When considering PM_2.5_ with NO_x_ and ozone simultaneously, Every increment of 1(μg/m^3^) in one-year PM_2.5_ exposure still showed a positive association with insomnia (1.624, 95% CI [1.570–1.681]). In addition, Every increment of 1(ppb) in one-year mean ozone exposure also showed a positive association with insomnia in the multi-pollutants model (odds ratio 1.198, 95% CI [1.094, 1.311] (Table 2). If we took the major chronic disease and roadway proximity from confounding factors, the association between air pollution and insomnia remained. Original data was not shown here.

**Table 2:** Adjusted odds ratios of insomnia for single pollutant and multiple pollutants by multiple logistic regression models for 5108 study subjects in Taipei City Hospital (N = 5108).

	OR (95% CI +)
**Single-pollutant**	
PM_2.5_	1.610 (1.562–1.660)*
Age (one-year increase)	1.020 (1.012–1.028)*
Gender (female/male)	1.216 (0.963–1.537)
Major chronic disease (≥ 1 disease/no diseases)	31.31 (19.83–49.42)*
Education (Non-college graduates /college graduates)	1.608 (1.201–2.154)*
Buffer 50 road area (100 m^2^ increase)	1.007 (0.997–1.017)
**Residence floor (reference:1–2 floor)**	
3-4floor	0.941 (0.712–1.244)
5-6floor	0.841 (0.626–1.130)
**O**_**3**_	1.116 (1.086–1.148)*
Age (one-year increase)	1.017 (1.011–1.023)*
Gender (female/male)	0.728 (0.617–0.860) *
Major chronic disease (≥ 1 disease/no diseases)	5.244 (3.712–7.408)*
Education (Non-college graduates /college graduates)	0.682(0.552–0.842)*
Buffer 50 road area (100 m^2^ increase)	1.009(1.002–1.017) *
**Residence floor (reference:1–2 floor)**	
3-4floor (reference:1–2 floor)	1.13 (1.017–1.256) *
> 5 floor(reference:3–4floor)	1.13 (1.017–1.256) *
NO_x_	1.025 (1.010–1.040)*
Age (one-year increase)	1.016 (1.010–1.022)*
Gender (female/male)	0.692 (0.587–0.816)*
Major chronic disease (≥ 1 disease/no diseases)	5.44 (3.851–7.684)*
Education (Non-college graduates /college graduates)	0.732(0.593–0.903)*
Buffer 50 road area (100 m^2^ increase)	1.005(0.997–1.012)
**Residence floor**	
3-4floor (reference:1–2 floor)	1.084(0.976–1.204)
> 5 floor(reference:3–4floor)	1.084(0.976–1.204)
**Multiple-pollutants**	
PM_2.5_ (μg/m^3^)	1.624 (1.570–1.681)*
NO_x_ (ppb)	0.997 (0.945–1.051)
O_3_ (ppb)	1.198 (1.094–1.311)*
Age (one-year increase)	1.020 (1.012–1.029)*
Gender (female/male)	1.136 (0.894–1.444)
Major chronic disease (≥ 1 disease/no diseases)	21.65 (14.14–33.15)*
Education (Non-college graduates /college graduates)	1.731 (1.288–2.326)*

*OR: odds ratio.

+ CI: confidence interval.

*Statistically significant, *p* < 0.05.

Single-pollutant: adjusted for age, gender, major chronic diseases, educational level, buffer 50 road area, and residence floor.

Multiple-pollutants: adjusted for age, gender, major chronic diseases, educational level.

### Subgroup analysis and sensitivity analysis

We further subgrouped our participants into those with major chronic diseases or those without major chronic diseases via stratified analysis. The without major chronic disease subgroup comprised 1026 non-insomniacs and 43 insomniacs. The odds ratio of one-year mean PM_2.5_ exposure[every increment of 1(μg/m^3^)] on insomnia was 1.351 (95% CI [1.218, 1.499]). In contrast, the with major chronic disease subgroup included 3,170 non-insomniacs and 869 insomniacs. The odds ratio of one-year mean PM_2.5_ exposure[every increment of 1(μg/m^3^)] on insomnia was 1.657 (95% CI [1.599, 1.717]). (Table3). Additionally, ozone also showed an increased risk of insomnia in the with major chronic diseases subgroup (odds ratio 1.190, 95% CI [1.084, 1.308], [every increment of 1 ppb]) than in the without major chronic diseases subgroup (odds ratio 1.166, 95% CI [0.833, 1.633]), and to a statistically significant degree in the formersubgroup. Age and gender modified effect of air pollution on insomnia were also assessed, which revealed that more risk of PM_2.5_ and O_3_ on insomnia for older people to a significant degree. Original data was not shown here.

**Table 3:** Comparison of the odds ratio of PM_2.5_, ozone, and NOx on insomnia with (N = 4039) and without (N = 1069) major chronic diseases estimated by stratified analysis.

	Without major diseases OR (95% CI)	With major diseases OR (95% CI)
**One-year exposure**		
Age	1.038 (1.016–1.060)*	1.016 (1.007–1.025)*
Gender (female/male)	0.841 (0.405–1.749)	1.193 (0.922–1.545)
Education (non-graduates/graduates)	1.752 (0.774–3.963)	1.745 (1.263–2.412)*
PM_2.5_	1.351 (1.218–1.499)*	1.657 (1.599–1.717)*
NO_x_	0.987 (0.817–1.194)	1.004 (0.950–1.061)
O_3_	1.166 (0.833–1.633)	1.190 (1.084–1.308)*

*Statistically significant: *p* < 0.05.

## Discussion

This study demonstrated a positive association between ambient PM_2.5_ and ozone exposure with the incidence of hypnotic-treated insomnia. After adjusting for sociodemographic factors (including age and gender) (Table 1), major chronic diseases (including psychiatric disease) (Supplementary Table), educational level, environmental factors (including residence floor level and traffic noise-indicated variables) (Table 2), PM_2.5_ maintained a positive association with insomnia.

In terms of air pollution, past studies have shown that exposure to higher concentrations of PM_2.5_ can affect sleep. Long-term exposure to PM_2.5_ was associated with poor sleep quality in rural China (mean: 72.3 µg/m^3^)^28^, and increased the probability of sleep disorders and sleep disorder symptoms in Chinese children (mean: 55.7 µg/m^3^)^26^. The average one-year PM_2.5_ exposure of our subjects with insomnia was 25.5 µg/m^3^, and that of non-insomnia subjects was 18.7 µg/m^3^. In our study, PM_2.5_ is positively correlated with the prevalence of insomnia, and PM_2.5_ levels are relatively high. Although our exposure concentration is lower than the above study, we still see similar results. The annual average PM_2.5_ exposure of the research subjects exceeded the upper limit of the air quality supervision of the Taiwan Environmental Protection Administration (annual average < 15 µg/m^3^) and the World Health Organization's standard. Furthermore, WHO improved the highest average annual emission level turned 10 μg/m^3^ into 5 μg/m^3^ for PM_2.5_ in 2021. Therefore, it is important to conduct research on the association between PM_2.5_ and insomnia at relatively low concentrations. Subgroup analysis revealed the interaction of PM_2.5_ with major chronic diseases. In this study, we assumed that every major chronic disease contributed to insomnia in the same degree. We also found that the effect of PM_2.5_ on insomnia was higher in patients with major chronic diseases than in those without a certain major chronic disease. This result implied that those with major chronic diseases were more susceptible to the influence of particulate matter on insomnia.

In addition to PM_2.5_, the Multiple pollutants model and stratified analysis in this study both showed ozone is another pollutant that is positively associated with insomnia. It implies that both primary and secondary air pollution played roles in insomnia in the urban environment. A past study has shown that increases in ozone concentration were associated with apnoea–hypopnoea index (AHI), a measure of the severity of SDB^27^. Other studies suggested that ozone exposure could affect the sleep–wake cycle in rats^34^. Several past studies have shown a link between NOx and sleep. One study found that long-term exposure to nitrogen dioxide was associated with an increased risk of poor sleep quality^28^. Both short- and long-term exposure to NO_2_ was associated with increased AHI, and this effect was caused by periods of rapid eye movement^35^. Therefore, it is worth exploring how urban air pollution affects sleep. Some studies have found that indoor air pollution can also affect sleep. When bedroom air quality was improved, sleep quality improved^36^. The common indoor air pollution is NO_x_, SO_2_, O_3_, CO, volatile and semi-volatile organic compounds (VOCs), PM, radon, and microorganism. However, it has been observed that part of the source of indoor air pollution is outdoor air pollution. The study in Taiwan found a similar increase in the concentration of PM_2.5_ and PM_10_ in both indoor and outdoor air during a dust storm event and interpreted the cause to be the extraction of outdoor air from their building's ventilation system^37^. Cluster analysis showed that the accumulation mode indoors is highly connected to the accumulation mode outdoors^38^. The study showed that the daily outdoor air change could explain about 75% of the daily indoor air change^39^. When the windows were open, the indoor PM_2.5_ concentration mainly came from outdoor-originating particles (over 92%), and when the windows were closed, about 54 ~ 63% of indoor PM_2.5_ concentration came from outdoor-originating particles^40^.

In demographic characteristics, one longitudinal study in Taiwan utilized the ICD-9-CM diagnostic and procedure codes from National Health Insurance Research Database; they reported that the prevalence and incidence of insomnia among those seeking healthcare increased from 2.17% in 2002 to 4.17% in 2009, and the prevalence increases with age from less than 12 years old to 80 years old. Individuals who were 50–64 years old (OR: 23.25) and above 65 years old (OR:24.70) were more likely to have insomnia than those younger than 20 years old; moreover, women were more likely to have insomnia than men (odds ratio 1.82; 95% confidence interval [CI], 1.79–1.86)^41^. In another study that investigated the community-dwelling senior citizens in Taipei City in 2009, insomnia syndrome was found in 41% of individuals, and it was more common in women than in men^42^. In our study, the mean age of our adult study population was 61.33 years, with a standard deviation 15.16. The insomnia prevalence of our hospital outpatients over 65 years old was 22.59%, and the risk of insomnia was higher for women than for men; these findings were reasonable compared with the results of previous studies. Insomnia is a patient-reported problem. In this study, we used the electrical records of diagnosis codes with hypnotic use as criterion for insomnia. This criterion restricted our insomniacs to individuals with severe insomnia symptoms such that they require medical help. To eliminate bias of hypnotic misuse by physicians or patient requests, we excluded those using hypnotics without an initial diagnosis of insomnia, which accounted for 24.57% of the original Taipei residents study subjects above 18 years old. To reduce confounding of air pollutant-caused anxiety and depressive symptoms or aggravating symptoms of any underlying chronic illness that may cause secondary insomnia, we adjusted major chronic diseases as well^5,43^. The cases with complete educational information were younger and comprised more males than the cases with missing educational information. Thus, the impact of air pollution on insomnia may be underestimated for our whole hospital-based study subjects. However, our hospital-based total population comprised older people compared with the general population of Taipei City (mean adult age, 47.47 years old)^44^. The prevalence of insomnia may be overestimated due to the old age of our study subjects. In previous studies, older people^45,46^, females^47^, and individuals with lower income and lower educational attainment^48^ were associated with more sleep complaints than younger people, males, and individuals with higher income and higher educational attainment. In our study, old age, females, and the educational level below college degree were associated with prevalent insomnia.

Environmental factors affect sleep and emotions. Previous studies using the Nordic prediction method to model nighttime traffic noise revealed that road traffic noise is associated with sleep disturbances or attention disorders^49,50^. In this study, the road area around the residence site, which was employed as a noise indicator, did not influence the risk of insomnia prevalence. The best-supported explanation may be that the road area in Taipei City does not reflect the actual traffic noises affected by traffic flow, vehicle speed, and noise barrier, and that people had the habit of keeping windows closed at night^51^. In addition, road area could be an indicator for both PM_2.5_ and noise.

The effects of pollutants on sleep remain unclear. The process of sleep is influenced by cytokines, such as tumor necrosis factor-α and interleukin-1β, and multiple neurotransmitters such as histamine, serotonin, and orexin^52,53^. Particles have been shown to translocate from the nose up the olfactory nerve into the brain, which is associated with increased brain inflammatory responses and changes in neurotransmitter levels^54^. Results from a previous study showed that healthy rats exposed to a single acute exposure to particulate matter demonstrated transiently increased plasma levels of adrenocorticotropic hormone and the glucocorticoid corticosterone, thereby confirming activation of the hypothalamic–pituitary–adrenal (HPA) axis^55^. The HPA axis is known to cause arousal and sleeplessness to humans and animals^56^. However, whether long-term exposure to ambient air pollution can increase the probability of HPA axis dysregulation has yet to be determined^55^.

Our study had several strengths. First, hospital-based data provided the complete addresses of each subject, so we could assess individual exposure to air pollutants. Second, many studies investigated the effect of ambient air pollutants on disability and mortality. Few analyzed the effects of air pollution on quality of life. Insomnia and sleeping pills use may have adverse effects, such as daytime drowsiness and dizziness or light-headedness^57^. These effects may influence working productivity and pose great economic costs^58,59^. Our study was the first to investigate the association between ambient air pollution and medical doctor-diagnosed insomnia with hypnotic use in an Asian population. However, our study had several limitations. First, participants could be lost to follow-up or visited another medical agency for insomnia treatment during the follow-up period, which may lead to outcome misclassification. Second, using electronic records of residential addresses and the nearest air monitor station database to estimate exposure may lead to non-differential misclassification because address changes and daytime activity changes may not reflect the actual exposure density. Third, we lacked the actual residential noise data. Fourth, some possible confounders, like body mass index, occupation, physical activity and smoking were not considered in our study due to lack or possible miscoding of the data. However, the economic activity and health resources were average in Taipei city. There was minimal health inequality problem in Taipei city. Fifth, further stud could consider involving the real time climate factors, like wind direction, wind speed, temperature and other environmental characteristics to mix land use regression model to better estimate our 1-year average air pollution exposure. Our findings warranted additional studies with individualized PM_2.5_, ozone, and NO_x_ exposure measurements in a cohort design to confirm the causal relationship between air pollution and insomnia.

## Conclusion and recommendation

We concluded that PM_2.5_ and ozone exposure are associated with insomnia and hypnotic use in Taipei. The effect of PM_2.5_ and ozone on insomnia were significant in people with major chronic diseases. Future health impact assessment on air pollution should include sleep disturbance as an important health outcome.

## Supplementary Information

Supplementary Information.

## Data Availability

The datasets generated and/or analyzed during the current study are not publicly available but are available from the corresponding author upon reasonable request.

## Abbreviations

SDB: Sleep disordered breathing
HIS: Health information system
ICD: International statistical classification of diseases and related health problems
DDD: Defined daily dose
GIS: Geographic information system
AHI: Apnoea–hypopnoea index
VOCs: Semi-volatile organic compounds
HPA: Hypothalamic–pituitary–adrenal
