# Supplementary material for: Association between ambient air pollution exposure and insomnia among adults in Taipei City
Source: Sci Rep. 2022 Nov 9;12:19064. doi: 10.1038/s41598-022-21964-0 (PMC9646727; doi:10.1038/s41598-022-21964-0)
Supplement: Supplementary file 1 — Supplementary Information. [file 41598_2022_21964_MOESM1_ESM.pdf]

Supplemental Material

Table S1 ICD-9 and ICD-10 codes of chronic major diseases

**Disease**	ICD-10	ICD-9
**Sleep disorder**		
Insomnia	G47.00,G47.09	780.52,780.54
Insomnia due to medical condition	G47.01	
Primary insomnia	F51.01	307.42, 307.41
Other insomnia not due to a substance or known physiological condition	F51.09	
**Psychiatric diseases**		
Manic disorder	F30	296.0x, 296.1x,296.81
Bipolar disorder	F31	296.4x,296.5x,296.6x
Major depressive disorder	F32,F33	296.2x,296.82,298,311
Persistent mood disorder	F34.x	296.99,301.1x
Unspecified mood disorder	F39	296.9
Anxiety		300, 300.4
**Cardiovascular disease**		
Coronary heart disease	I21,I22	410-414
Congestive heart failure	I50.1-I50.9	428.0-428.9

**Neurological disease**		
Cerebrovascular accident	I60, I61, I62, I63, I64, I65, I66, G450, G451, G452, G454, G458, G459, G46, I670, I671, I672, I674, I675, I676, I677 I678, I679, I681, I682, I688, I69	430-438
Dementia	F00, F01, F02,F03, F051	290
**Pulmonary disease**	J41, J42, J44, J43, J45, J46, J47, J67, J44, J60, J61,J62, J63, J66, J64, J65	491, 492, 493, 494, 495, 496,500, 501, 502, 503, 504, 505
**Peptic ulcer disease and reflux esophagitis**	K25, K26, K27, K28	531, 532, 533, 534 530.11,530.81
**Diabetes**	E102, E103,E104, E109, E119, E139, E149, E101, E111, E131, E141, E105, E115,E135, E145 E112, E132, E142, E113, E133, E143 E114,E134, E14	250
**Renal disease**	N03, N052, N053, N054, N055, N056, N072, N073, N074, N01, N18, N19, N25	582, 5830, 5831, 5832, 5835,5836, 5837, 5834, 585,586,588
**Malignancy**	C	140-208

Table S2 Diseases of the study subjects without chronic major diseases (N=1,069)

Diseases	ICD 9 codes	Number of study subjects
Eye and adnexa system disease	360-379	239
Hyperlipidemia	272.0-272.4	160
Disease of esophagus stomach, duodenum and intestine	530, 535-537,555-558,564,009	141
Hypertension	401-405	138
Acute upper respiratory tract infection	460-466	126
Vaccination	V	94
Osteoarthritis	715	68
Cardiac diseases	424-429	67
Hyper-or hypothyroidism	240-246	56
Skin diseases	690-698	42
Upper respiratory tract diseases	470-478	35

Table S3 Study anxiolytics and hypnotic drugs and defined daily dose (DDD)

Drug	DDD (mg)
**Benzodiazepines**	
Alprazolam	1
Brotizolam	0.25
Bromazepam	10
Clonazepam	8
Diazepam	10
Estazolam	3
Flunitrazepam	1
Flurazepam	30
Fludiazepam	0.75
Lorazepam	0.5
Nordazepam	15
Oxazepam	50
Triazolam	0.25
**Z-drugs**	
Zaleplon	10
Zolpidem	10
Zolpiclone	7.5

Table S4 Comparison of age, gender, and education of the insomniacs and non-insomniacs of the outpatients aged above 18 years old and living in Taipei City (N=28,232)

**Characteristics**	**Non-insomniacs**	**Insomniacs**	**Total**	**P value**
**Number of patients(%)**	23,364 (83)	4868 (17)	28,232	
**Age, year**				<0.0001
**Mean (SD)**	61.69 (16.67)	67.94 (14.04)	62.76(16.42)	
**Gender**				<0.0001
Female, n(%)	11,102 (48)	2,830 (58)	13,932 (49)	
Male, n(%)	12,262 (52)	2,038 (42)	14,300 (51)	
**Education**				<0.0001
Non-college(%)	2,993 (71)	743 (81)	3,736 (73)	
College(%)	1,203 (29)	169 (19)	1,372 (27)	
**Residence floor , n (%)**				0.58723
1-2	5,680 (30)	1,179 (30)	6,859 (30)	
3-4	7,297 (39)	1,493 (38)	8,790 (39)	
≧5	5,942 (31)	1,205 (31)	7,147 (31)	

Table S5 Comparison of age, gender, and insomnia status between 5,108 cases with complete educational information and excluded 23,124 cases with missing educational information

**Characteristics**	**Education information complete**	**Education information missing**	**Total**	**P-value**
**Number**	5108	23124	28232	
**Age year, mean(SD)**				<0.0001
	61.33 (15.16)	63.08 (16.67)	62.76(16.42)	
**Gender**				
Female(%)	2,393 (47)	11,539 (50)	13,932 (49)	<0.0001
Male(%)	2,715 (53)	11,585 (50)	14,300 (51)	
**Insomnia state**				0.2011
Insomniacs(%)	912 (18)	3,956 (17)	23,364 (83)	
Non-insomniacs(%)	4,196 (82)	19,168 (83)	4,868 (17)	
